# *GHRL* Gene Leu72Met Polymorphism and Type 2 Diabetes Mellitus: A Meta-Analysis Involving 8,194 Participants

**DOI:** 10.3389/fendo.2019.00559

**Published:** 2019-08-08

**Authors:** Yan-yan Li, Xin-zheng Lu, Xin-xing Yang, Hui Wang, Hong-yu Geng, Ge Gong, Yi-yang Zhan, Hyun Jun Kim, Zhi-jian Yang

**Affiliations:** ^1^Clinical Research Center, First Affiliated Hospital of Nanjing Medical University, Nanjing, China; ^2^Department of Gerontology, First Affiliated Hospital of Nanjing Medical University, Nanjing, China; ^3^Department of Cardiology, First Affiliated Hospital of Nanjing Medical University, Nanjing, China; ^4^Department of Intensive Care Unit, Baoding First Center Hospital, Baoding, China; ^5^Department of Gerontology, Nanjing General Hospital of Nanjing Military Command, Nanjing, China; ^6^Department of Physiology, University of Cincinnati, Cincinnati, OH, United States

**Keywords:** *GHRL*, Leu72Met, polymorphism, type 2 diabetes mellitus, T2DM

## Abstract

**Background:** Although many studies indicate a positive correlation between *GHRL* gene Leu72Met polymorphism and an increased susceptibility to type 2 diabetes mellitus (T2DM), inconsistencies between independent studies still remain.

**Objective:** Considering the inconsistencies between them, we have performed the current meta-analysis study. The objective of this study is to better examine the correlation of the *GHRL* gene Leu72Met polymorphism and T2DM.

**Methods:** The current meta-analysis, involving 8,194 participants from 11 independent studies, was performed. A fixed effect model was used to evaluate the pooled odds ratios (ORs) and the corresponding 95% confidence intervals (95% CIs).

**Results:** A significant association was found between T2DM and *GHRL* gene Leu72Met polymorphism under recessive (OR: 1.33, 95% CI: 1.01–1.76, *P* = 0.04), and homozygous genetic models (OR: 1.34, 95% CI: 1.01–1.78, *P* = 0.04) in the whole population. The correlation was more distinct in our subgroup analysis of the Chinese population under recessive (OR: 1.52, 95% CI: 1.07–2.15, *P* = 0.02), dominant (OR: 1.70, 95% CI: 1.38–2.10, *P* < 0.00001), additive (OR: 1.16, 95% CI: 1.02–1.33, *P* = 0.02), and homozygous genetic models (OR: 1.54, 95% CI: 1.07–2.20, *P* = 0.02).

**Conclusions:** In short, *GHRL* gene Leu72Met polymorphism was significantly correlated with increased T2DM risk, particularly in the Chinese population. Individuals carrying the Met72 allele of *GHRL* Leu72Met gene polymorphism, particularly those of Chinese ancestry, may be more susceptible to developing T2DM disease.

## Introduction

Type 2 diabetes mellitus (T2DM) is characterized by hyperglycemia in the context of insulin resistance and impaired insulin secretion. Its long-term complications include abnormal protein and lipid metabolism and multisystem damage (1). T2DM is a multi-genic disease caused by the synergistic interactions between hereditary and environmental factors. Recently multiple genes determining the presence of T2DM have been reported as *ATP binding cassette transporter 1 (ABCA 1)* (2), β*-defensin 1* (3), *5- lipoxygenase (ALOX5)* (4), *solute carrier family 30 member 8 (SLC30A8)* (5), *ADIPOQ, KCNJ11, TCF7L2* (6), *superoxide dismutase 2 (SOD2)* (7), *interleukin-10 (IL-10)* (8), *paraoxonase 2 (PON2)* gene (9), and *GHRL (Ghrelin)* (10). While hereditary factors play an important role in the T2DM development, clarification of their exact mechanisms depend on the identification of T2DM susceptibility genes.

*GHRL (Ghrelin)* is a promising susceptibility gene for T2DM. First discovered by Kojima in 1999, the *GHRL* gene encodes for a 28 amino acid brain-gut peptide hormone, ghrelin (10). Although ghrelin is primarily synthesized and secreted by X/A-like oxyntic cells of the stomach, it is also produced within the hypothalamus and pancreas (11). Ghrelin is also synthesized by other tissues, such as small intestine, kidney, heart, adipose tissue, and so on (12). As the endogenous ligand of the growth-hormone secretagogue receptor (GHS-R), ghrelin promotes adenohypophysis to release growth hormone, but also has roles in appetite regulation, fat accumulation, gut motility, and energy balance (13). Additionally, ghrelin may play a role in the blood glucose and insulin regulation as decreased plasma ghrelin levels was associated with T2DM (14).

The *GHRL* gene, located in 3p25-26, contains 4 exons and 3 introns. The four exons encode preproghrelin with 117 amino acids, of which, the first and second encode mature ghrelin with 28 amino acids (15). Most of *GHRL* polymorphisms studied to date are located in the promoter and in the coding regions of the gene and some of them have implications for gene activity (16). The polymorphism most strongly associated with T2DM is the Leu72Met polymorphism (+408C>A) and by switching cytosine (C) in the second exon to adenine (A), the mutation replaces the leucine (Leu) at the 72nd amino acid with a methionine (Met).

Though many studies examining the association between the *GHRL* gene Leu72Met polymorphism and T2DM have been conducted, the conclusions of these studies have often been contradictory. In 2006, Mager et al. reported that Finnish subjects with the Leu72Leu genotype had a decreased risk for T2DM development and that the Met72 allele might be a contributory factor to T2DM (17). In 2012, Wang et al. reproduced the result in China (18). On the contrary, in 2008, Berthold et al. observed that German subjects with Met72+ genotypes had a significantly reduced risk of T2DM (OR 0.63, 95% CI 0.42–0.95, *P* = 0.026) (19). In 2006, Choi et al. reported that *GHRL* gene Leu72Met polymorphism was not found to be significantly associated with susceptibility to T2DM in the Korean population (20).

The current meta-analysis including 4,299 T2DM cases and 3,895 controls was carried out to evaluate the association of T2DM and *GHRL* Leu72Met gene polymorphism (Supplement S1).

## Materials and Methods

### The Eligible Publications Hunt and Inclusion Standards (21)

We searched databases, such as the China National Knowledge Infrastructure, the China Biological Medicine Database, Pubmed, Embase, and the Web of Science, using the terms, “*GHRL*,” “Leu72Met,” “type 2 diabetes mellitus,” and “polymorphism.” The search deadline was on December 17, 2018 and the publication date of the resultant studies ranged from 2005 to 2012.

The selected studies had to conform to the following inclusion criteria: (a) assessment of the association between T2DM and *GHRL* Leu72Met gene polymorphism. (b) fasting plasma glucose (FPG) is used in the diagnosis of T2DM as proposed by the American Diabetes Association (2005). The FPG level ≥7.0 mmol/L or the 2 h plasma glucose of oral glucose tolerance test ≥11.1 mmol/L. No genetic relationships are present between the participants of the study. (c) Adopted data is derived from case-control or cohort studies published in the official journals or postgraduate dissertations. (d) In the individual studies, the control group genotype member should follow the Hardy-Weinberg equilibrium (HWE).

### Data Extraction (21)

The data present in the studies was extracted according to classic protocol. Several investigators performed the meta-analysis. Two investigators retrieved the studies in duplicate and a third served as the arbitrator to settle differences between the two investigators and helped form a consensus. The excluded researches included those that failed to satisfy inclusion criteria, were repeated in other publications present in the analysis, or supplied inadequate information. Same data presented in different papers, but derived from the same group of authors was used only once. Items, such as the name of the first author, publication date, research region, genotypes numbers, and a whole number of subjects were included in the extracted data.

### Statistical Analyses

Four genetic models were used in the meta-analysis: recessive (MM vs. LL+LM), dominant (LM+MM vs. LL), homozygous (MM vs. LL), and additive (M vs. L) genetic models. The association of *GHRL* gene Leu72Met polymorphism and T2DM was analyzed using odds ratios (ORs) and their corresponding 95% confidence intervals (CIs). The heterogeneity among the studies was calculated by the chi-square-based *Q*-test (*P* < 0.05) (22). If heterogeneity were present, the random-effect model using the DerSimonian and Laird method would be employed (23). But in the case that heterogeneity was not detected, the fixed-effect model using the Mantel-Haenszel method would be adopted (24). The pooled OR was estimated by *Z*-test (*P* < 0.05).

The Fisher's exact test was used to assess the HWE (*P* < 0.05) and the funnel plot, to estimate potential publication bias. Egger's linear regression test on the natural logarithm scale of the OR was used to evaluate the funnel plot symmetry (*P* < 0.05) (25). The Stata 11.0 software (StataCorp, College Station, TX, USA) was used to perform the statistical analyses.

## Results

### Studies and Populations

Of the 19 manuscripts produced by the search 11 satisfied the inclusion criteria. Among the eight discarded studies, three papers were repeated publications, two were reviews, and the remaining three were not related to the topic in question. None of the studies deviated from the HWE. The data listed in Table 1 was drawn from 4,299 T2DM patients and 3,895 controls (1, 17, 19, 20, 26–32) (Supplement S2). The participants of the studies represent six countries: China, Denmark, Finland, France, Germany, and Korea. For our analysis, participants from Denmark, Finland, France, Germany, and Korea were categorized into the non-Chinese subgroup. Within the Chinese subgroup, participants hailed from five different provinces: Beijing, Shanghai, Guangdong, Heilongjiang, and Gansu.

**Table 1:** Characteristics of the investigated studies of the association between type 2 diabetes mellitus (T2DM) and *GHRL* gene Leu72Met (L72M) polymorphism.

**References**	**Region**	**T2DM**			**Control**			**Matching criteria**	**Sample size(T2DM/control)**
		**LL**	**LM**	**MM**	**LL**	**LM**	**MM**		
Jiang et al. (26)	China	151	96	5	55	28	0	Ethnicity	252/83
Xu and Xiang (27)	China	143	57	2	251	80	2	Age, sex, ethnicity	202/333
Cui et al. (1)	China	71	25	6	68	27	0	Age, sex, ethnicity	102/95
Xiang et al. (28)	China	272	37	7	179	21	2	Age, sex, ethnicity	316/202
Liu et al. (29)	China	463	336	65	472	353	52	Age, sex, ethnicity	864/877
Larsen et al. (30)	Denmark	455	71	2	194	33	2	Age, sex, ethnicity	528/229
Choi et al. (20)	Korea	518	215	25	429	185	22	Sex, ethnicity	758/636
Kim et al. (31)	Korea	133	65	8	54	23	3	Age, sex, ethnicity	206/80
Mager et al. (17)	Finland	37	12	2	144	40	3	Age, sex, ethnicity	51/187
Berthold et al. (19)	German	377	40	3	364	65	1	Age, sex, ethnicity	420/430
Garcia et al. (32)	France	523	75	2	633	108	2	Ethnicity	600/743

*T2DM, type 2 diabetes mellitus. Polymerase chain reaction-restriction fragment length polymorphism genotyping method and Case-control study design were adopted in the above studies*.

### Combined Analyses

**Figure 1:**
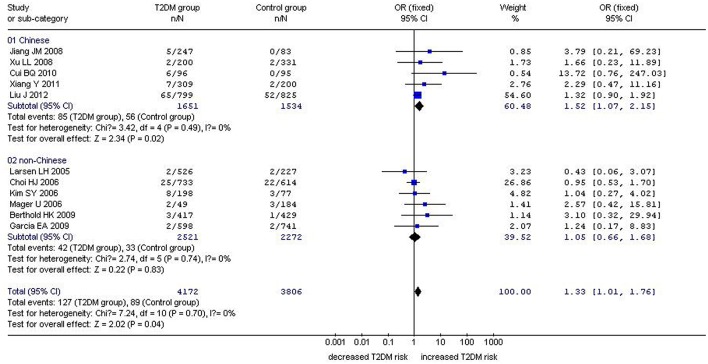
Forest plot of T2DM associated with *GHRL* gene Leu72Met (L72M) polymorphism under a recessive genetic model (MM vs. LM+LL). Under the recessive genetic model, a significant correlation between the *GHRL* Leu72Met gene polymorphism and T2DM was found (OR: 1.33, 95% CI: 1.01–1.76, *P* = 0.04). A more pronounced correlation between them was found (OR: 1.52, 95% CI: 1.07–2.15, *P* = 0.02) in the Chinese population. No significant correlation was detected in the non-Chinese population (OR: 1.05, 95% CI: 0.66–1.68, *P* = 0.83).

**Figure 2:**
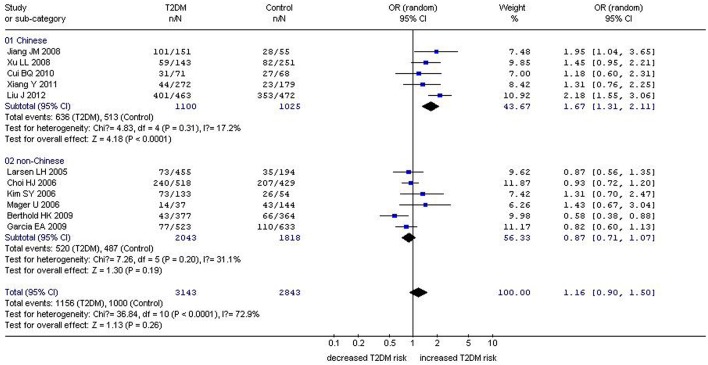
Forest plot of T2DM associated with *GHRL* gene Leu72Met (L72M) polymorphism under a dominant genetic model (LM+MM vs. LL). Under the dominant genetic model, no significant correlation was found (OR: 1.16, 95% CI: 0.90–1.50, *P* = 0.26). A significant correlation between them was found (OR: 1.70, 95% CI: 1.38–2.10, *P* < 0.00001) in the Chinese population. No significant correlation was detected in the non-Chinese population (OR: 0.87, 95% CI: 0.71–1.07, *P* = 0.19).

**Figure 3:**
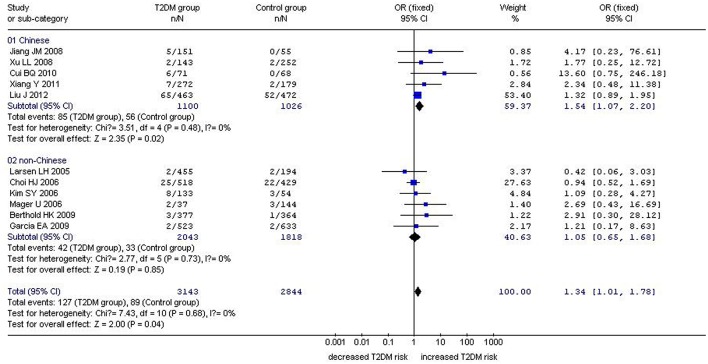
Forest plot of T2DM associated with *GHRL* gene Leu72Met (L72M) polymorphism under a homozygous genetic model (MM vs. LL). Under the homozygous genetic model, a significant correlation between the *GHRL* Leu72Met gene polymorphism and T2DM was found (OR: 1.34, 95% CI: 1.01–1.78, *P* = 0.04). A more pronounced correlation between them was found (OR: 1.54, 95% CI: 1.07–2.20, *P* = 0.02) in the Chinese population. No significant correlation was detected in the non-Chinese population (OR: 1.05, 95% CI: 0.65–1.68, *P* = 0.85).

**Figure 4:**
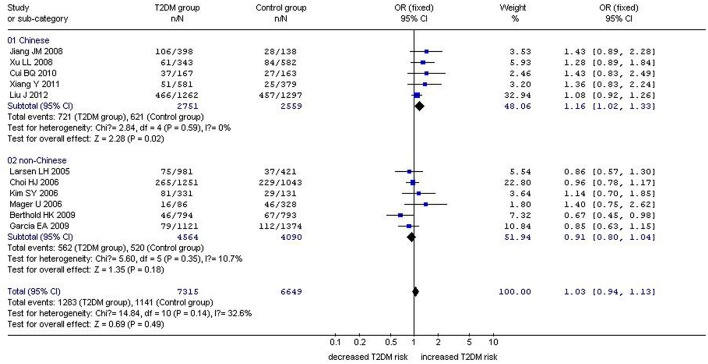
Forest plot of T2DM associated with *GHRL* gene Leu72Met (L72M) polymorphism under an additive genetic model (M vs. L). Under the additive genetic model, no significant correlation was found (OR: 1.03, 95% CI: 0.94–1.13, *P* = 0.14). A significant correlation between them was found (OR: 1.16, 95% CI: 1.02–1.33, *P* = 0.02) in the Chinese population. No significant correlation was detected in the non-Chinese population (OR: 0.91, 95% CI: 0.80–1.04, *P* = 0.18).

In our analysis of the whole population, we found a significant correlation between the *GHRL* Leu72Met gene polymorphism and T2DM under recessive (OR: 1.33, 95% CI: 1.01–1.76, *P* = 0.04) and homozygous genetic models (OR: 1.34, 95% CI: 1.01–1.78, *P* = 0.04). No significant correlation was found under dominant (OR: 1.16, 95% CI: 0.90–1.50, *P* = 0.26) or additive genetic models (OR: 1.03, 95% CI: 0.94–1.13, *P* = 0.14). In our subgroup analysis of the non-Chinese population, we found no significant correlation was detected under recessive (OR: 1.05, 95% CI: 0.66–1.68, *P* = 0.83), dominant (OR: 0.87, 95% CI: 0.71–1.07, *P* = 0.19), homozygous (OR: 1.05, 95% CI: 0.65–1.68, *P* = 0.85), and additive genetic models (OR: 0.91, 95% CI: 0.80–1.04, *P* = 0.18; Table 2, Figures 1–4). On the other hand, our subgroup analysis of the Chinese population found a more pronounced correlation between the *GHRL* Leu72Met gene polymorphism and T2DM under recessive (OR: 1.52, 95% CI: 1.07–2.15, *P* = 0.02), dominant (OR: 1.70, 95% CI: 1.38–2.10, *P* < 0.00001), additive (OR: 1.16, 95% CI: 1.02–1.33, *P* = 0.02), and homozygous (OR: 1.54, 95% CI: 1.07–2.20, *P* = 0.02).

**Table 2:** Summary of meta-analysis of association of type 2 diabetes mellitus (T2DM) and *GHRL* gene Leu72Met (L72M) polymorphism.

**Genetic model**	**Pooled OR (95% CI)**	***P*-value**	**Literature number**	**T2DM size**	**Control size**	***P*_**heterogeneity**_ (*I*^**2**^%)**
Recessive genetic model	1.33 (1.01–1.76)	0.04*	11	4,299	3,895	0.70 (0%)
Subgroup 1: Chinese	1.52 (1.07–2.15)	0.02*	5	1,736	1,590	0.38 (0%)
Subgroup 2: non-Chinese	1.04 (0.64–1.69)	0.87	6	2,563	2,305	0.74 (0%)
Dominant genetic model	1.16 (0.90–1.50)	0.26	11	4,299	3,895	<0.0001* (72.9%)
Subgroup 1: Chinese	1.70 (1.38–2.10)	<0.00001*	5	1,736	1,590	0.31 (17.2%)
Subgroup 2: non-Chinese	0.87 (0.71–1.07)	0.19	6	2,563	2,305	0.20 (31.1%)
Homo genetic model	1.34 (1.01–1.79)	0.05*	11	4,299	3,895	0.59 (0%)
Subgroup 1: Chinese	1.54 (1.07–2.20)	0.02*	5	1,736	1,590	0.48 (0%)
Subgroup 2: non-Chinese	1.05 (0.65–1.68)	0.85	6	2,563	2,305	0.73 (0%)
Additive genetic model	1.03 (0.94–1.13)	0.49	11	4,299	3,895	0.14 (32.6%)
Subgroup 1: Chinese	1.16 (1.02–1.33)	0.02*	5	1,736	1,590	0.59 (0%)
Subgroup 2: non-Chinese	0.91 (0.80–1.04)	0.18	6	2,563	2,305	0.35 (10.7%)

* *P < 0.05. CI, confidence interval; OR, odds ratio; T2DM size, the total number of T2DM cases; control size, the total number of control group; homo genetic model, homozygous genetic model*.

### Bias Diagnostics

**Figure 5:**
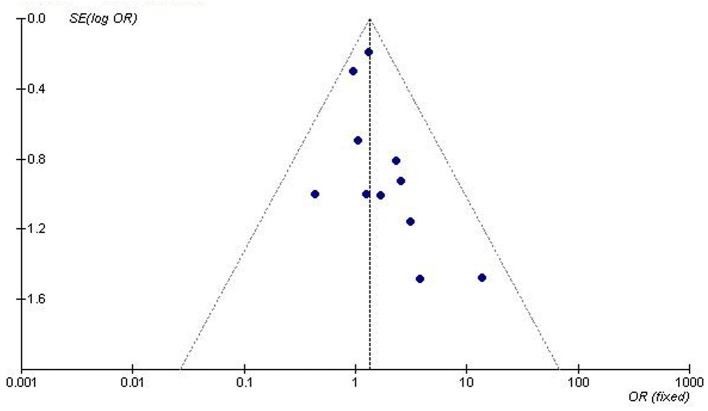
Funnel plot for studies of the association of T2DM associated with *GHRL* gene Leu72Met (L72M) polymorphism under a recessive genetic model (LM+MM vs. LL). The dot distribution in the funnel plot is symmetrical which imply no publication bias is present in the current meta-analysis by using the recessive genetic model. The horizontal and vertical axis correspond to the OR and confidence limits. OR, odds ratio; SE, standard error.

We used the funnel plot and Egger's test to assess for publication bias among the individual studies. The symmetry of the funnel plot (Figure 5) and the lack of significant difference detected by the Egger's test imply no publication bias is present in the current meta-analysis by using the recessive genetic model (T = −0.61, *P* = 0.563).

## Discussion

In the current meta-analysis, we observed a significant correlation in the whole population between the *GHRL* gene Leu72Met polymorphism and T2DM under recessive (OR: 1.33) and homozygous genetic models (OR: 1.34). While correlation in the non-Chinese population was not significant under any of the genetic models (*P* > 0.05), correlation in the Chinese population was significant under all four genetic models—recessive (OR: 1.52), dominant (OR: 1.70), homozygous (OR: 1.54), and additive genetic models (OR: 1.16). Though the Met72 allele of *GHRL* Leu72Met gene polymorphism has no significant effect on the T2DM susceptibility in the non-Chinese population, data suggested that the mutation might be strongly associated with the increased T2DM risk within the Chinese people. The significant difference between the two populations might be attributable to the difference in region and ethnicity.

Ghrelin has been shown to takes part in glucose metabolism which could promote insulin production in rats (13). Ghrelin secretion can also be inhibited by insulin. For example, obese individuals with elevated levels of plasma glucose had decreased ghrelin levels due to hyperinsulinemia. It has also been observed that the plasma ghrelin concentrations were negatively correlated with fasting insulin level, hypertension, insulin resistance and T2DM prevalence (33, 34). Levels of circulating ghrelin were even reduced in the healthy offspring of T2DM (35). Animal experiments and clinical researches have shown that the low ghrelin level could serve as the prediction indicator for obesity and T2DM (14). In 2002, Saad et al. reported that ghrelin administration reduced insulin secretion in human (36).

Although the *GHRL* gene Leu72Met polymorphism does not alter the sequence of mature ghrelin, changes to mRNA stability or protein synthesis may contribute to T2DM development by affecting ghrelin secretion and activity. In 2008, Zavarella et al. observed a significant decreasing trend from Leu/Leu to Leu/Met and to Met/Met homozygous subjects in fasting insulin levels, triglycerides, and HOMA-IR index and an increase trend in ghrelin levels. Their observations suggested that the Met72 variant might play a protective role in modulating insulin resistance. They also speculated that an unknown Met72 functional variant in linkage disequilibrium could increase the ghrelin levels and probably improve insulin sensitivity (37).

Ghrelin is present in the bloodstream in two major forms: desacyl and acylated ghrelin (38). Under physiological conditions, the ratio of acylated/des-acyl ghrelin ranges from 1:3 to 1:4, but this ratio varies under pathological conditions, including T2DM. In this regard, several reports have found that insulin resistance is associated with a reduction in circulating des-acyl ghrelin, while acylated ghrelin levels are increased (39, 40) or unchanged (41, 42). In addition, opposite effects of ghrelin isoforms on hepatic glucose metabolism have been found with des-acyl form suppressing hepatocyte glucose release as well as antagonizing the acylated ghrelin-induced increase in hepatic glucose output *in vitro* (43). In 2004, Vivenza et al. reported that *GHRL* gene polymorphisms (Arg51Gln, Leu72Met, and Gln90Leu), do not have a relevant impact in the secretion of total and acylated ghrelin (44).

Liao et al. in 2013 reported a protective effect from T2DM in the Caucasians population, but no correlation between the genetic mutation and T2DM in the overall population (45). As only three studies were included in the Caucasian subgroup and six of the whole population, this finding may be inaccurate. A 2012 meta-analysis by Liu et al. found no correlation from a relative large Chinese population, but also found that FPG levels of T2DM patients with Leu72Leu genotype were significantly higher than that of other variant genotypes (Leu72Met and Met72Met) (29). Though Liu's data alone did not find statistically significant correlation, once their work was incorporated into the current meta-analysis, the final results indicated a significant correlation between them. Because of the relatively limited sample size in Liu's individual study compared to that of the current meta-analysis, it is possible that the relationship between *GHRL* gene Leu72Met polymorphism with T2DM susceptibility was simply undetected by Liu's individual study. In 2012, Wang et al. also performed a similar meta-analysis of the Chinese population and came to a similar conclusion as the current meta-analysis (18). Because two of the five studies in Wang's paper were conducted by the same authors and had significant overlaps in data, their meta-analysis may not be as sound as the one presented here.

There are inevitably some limitations in the current meta-analysis. The current meta-analysis still lacks large-scale studies on the correlation between the *GHRL* gene Leu72Met polymorphism and T2DM. The ghrelin level was influenced not only by the *GHRL* gene Leu72Met polymorphism, but also by other gene polymorphism as Arg52Gln, and unhealthy dietary habits (46). As the genetic susceptibility model of T2DM was predominantly minor gene model, the genetic heterogeneity caused the T2DM susceptibility being influenced by races, regions, environment, and ancestry (47).

Finally, *GHRL* gene Leu72Met polymorphism was significantly associated with the increased T2DM risk, especially among the Chinese people. Individuals carrying the Met72 allele of *GHRL* Leu72Met gene polymorphism, particularly those of Chinese ancestry, may be more susceptible to developing T2DM disease. The above conclusion may open a novel window in guiding us to formulate a new T2DM therapy, especially in the Chinese T2DM population. Considering the limitations of this study, more large-scale researches are still necessary to clarify the conclusion in the long run.

## Disclosure

The authors report no relationships that could be construed as a conflict of interest.

## Author Contributions

YL: conceived and designed the experiments, statistical analyses, and paper writing. YL, GG, HG, and HW: performed the experiments. YL, XY, and XL: analyzed the data. YL and HW: contributed reagents, material, and analysis tools. YL and HK: wrote the manuscript. YL and XL: reference collection and data management. YL, YZ, and ZY: study design.

### Conflict of Interest Statement

The authors declare that the research was conducted in the absence of any commercial or financial relationships that could be construed as a potential conflict of interest.
