# Supplementary material for: GHRL Gene Leu72Met Polymorphism and Type 2 Diabetes Mellitus: A Meta-Analysis Involving 8,194 Participants
Source: Front Endocrinol (Lausanne). 2019 Aug 8;10:559. doi: 10.3389/fendo.2019.00559 (PMC6694458; doi:10.3389/fendo.2019.00559)
Supplement: Supplementary file 2 [file Table_2.DOC]

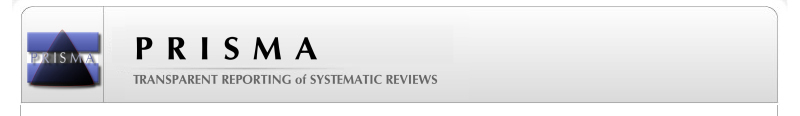
**PRISMA 2009 Flow Diagram**

**Screening**

**Included**

**Eligibility**

**Identification**

Records identified through database searching
(n =19 )

Additional records identified through other sources
(n =0 )

Records after duplicates removed
(n =16)

Records screened
(n =14 )

Records excluded for review characteristic
(n =2 )

Full-text articles assessed for eligibility
(n =14)

Full-text articles excluded for deviation from HWE (n =0 )

Studies included in qualitative synthesis
(n =11)

Records excluded for no association with ghrelin gene Leu72Met polymorphism or T2DM

(n =3 )

Records excluded for repeated publication
(n = 3 )
